# Supplementary material for: Unbalanced dendritic inhibition of CA1 neurons drives spatial-memory deficits in the Ts2Cje Down syndrome model
Source: Nat Commun. 2019 Nov 1;10:4991. doi: 10.1038/s41467-019-13004-9 (PMC6825203; doi:10.1038/s41467-019-13004-9)
Supplement: Supplementary file 1 — Supplementary Information [file 41467_2019_13004_MOESM1_ESM.pdf]

Unbalanced dendritic inhibition of CA1 neurons drives spatial-memory deficits in the Ts2Cje Down syndrome model

Valbuena et al.

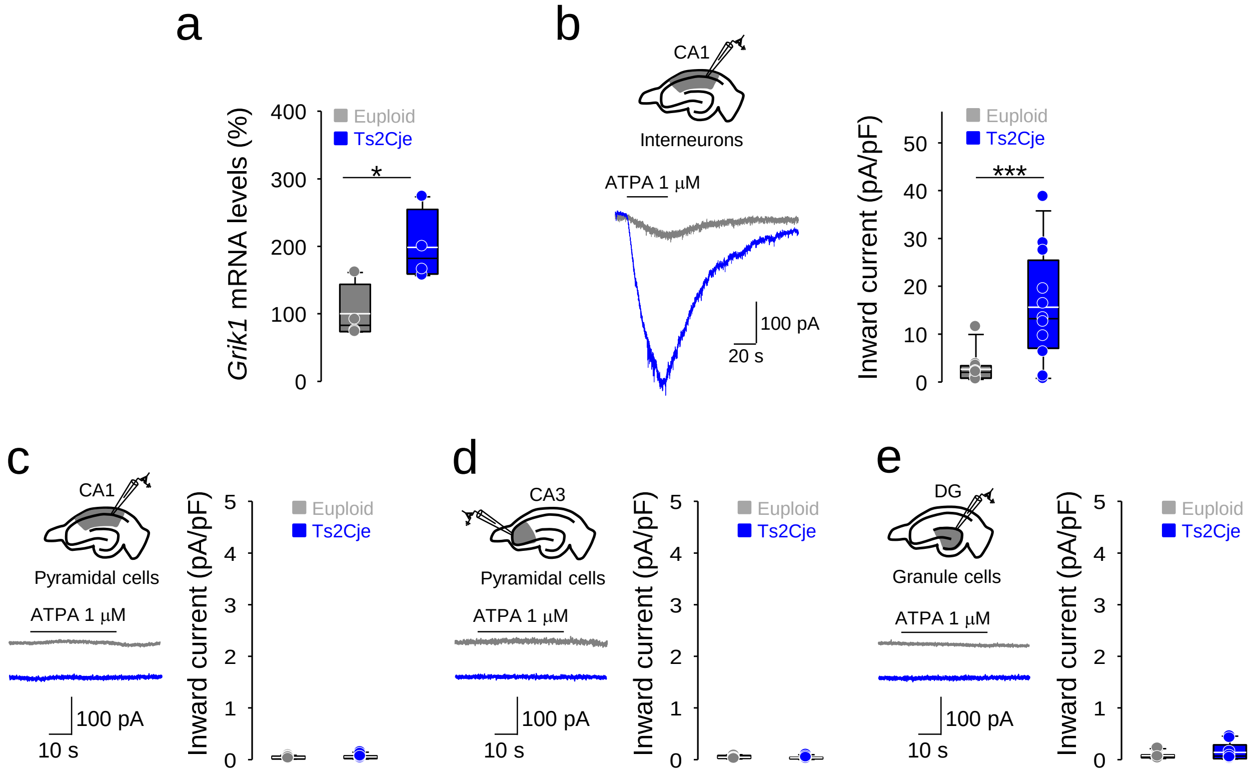

Supplementary Figure 1. Increased *Grik1* mRNA and GluK1 protein in Ts2Cje mice. a, Real-time qPCR quantification of *Grik1* mRNA levels in the hippocampus of Euploid and Ts2Cje mice. Levels of expression were normalized to those of Euploid animals. **b**, Left, representative traces of currents activated by perfusion of 1 μM ATPA to CA1 interneurons. Right, box plot representing increased GluK1-mediated current density induced by application of 1 μM ATPA in CA1 interneurons. **c-e**, Schematic illustrations of the area recorded and representative traces of ATPA-induced responses (left) in CA1 pyramidal neurons (c), CA3 pyramidal neurons (d) and granule dentate gyrus cells (e), illustrating the absence of ATPA-induced currents in these cells. Box plots representing the average from several neurons from different animals are shown on the right. **d**, Left, schematic view of the area recorded and representative ATPA-induced responses in CA3 pyramidal neurons. For detailed data values and statistics see Supplementary Table 1. **p* < 0.05, ****p* < 0.005.

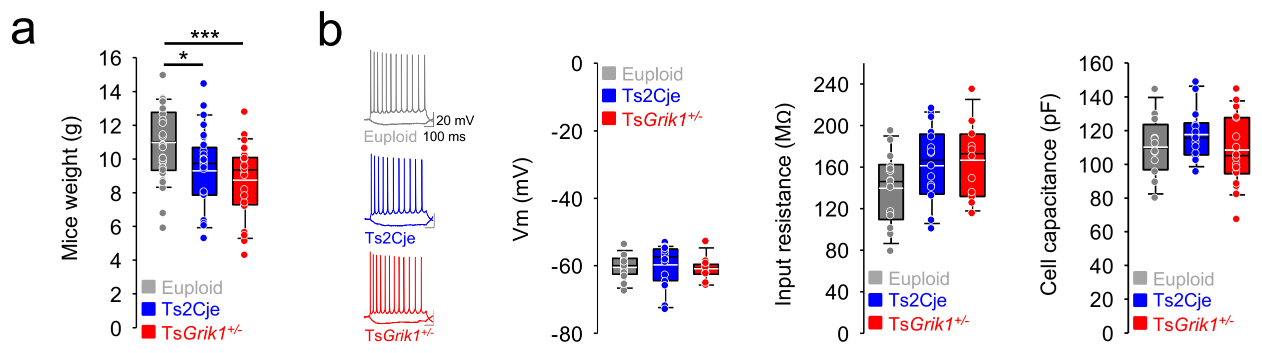

Supplementary Figure 2. Reduced weight but unaltered CA1 pyramidal cell parameters in Ts2Cje and Ts*Grik1*$^{+/-}$ mice. a, Mice weight measured at P19-P21. Ts2Cje and Ts*Grik1*$^{+/-}$ mice presented reduced weight in comparison to euploid littermates. **b**, Left, representative traces of voltage responses to -50 pA and +300 pA current injections in CA1 pyramidal cells. Box plots representing resting membrane potential (middle left), input resistance (middle right) and capacitance (right). For detailed data values and statistics see Supplementary Table 1. *$p < 0.05$, ***$p < 0.005$.

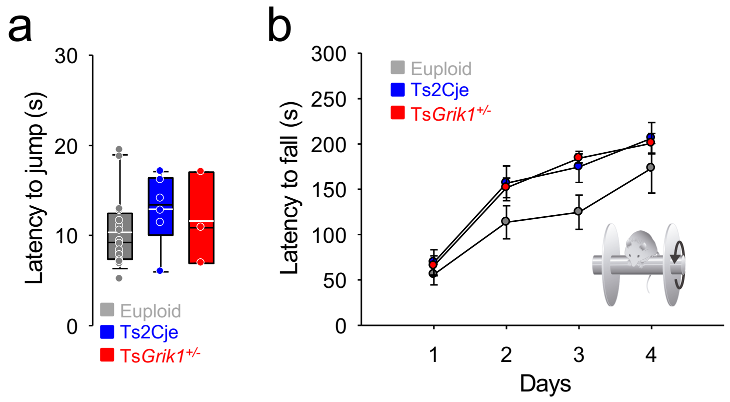

Supplementary Figure 3. Unaltered pain sensitivity and motor performance in Ts2Cje and Ts*Grik1*$^{+/-}$ mice. a, Box plot illustrating the latency to jump in the hot plate test. Trisomic animals (Ts2Cje and Ts*Grik1*$^{+/-}$) showed similar latencies in comparison to Euploid littermates, suggesting unaltered pain sensitivity. **b**, Time course of the latency to fall from the rotarod device across days. No differences where found in Ts2Cje and Ts*Grik1*$^{+/-}$ mice in comparison to Euploid littermates, indicating unaltered motor performance. For detailed data values and statistics see Supplementary Table 1.

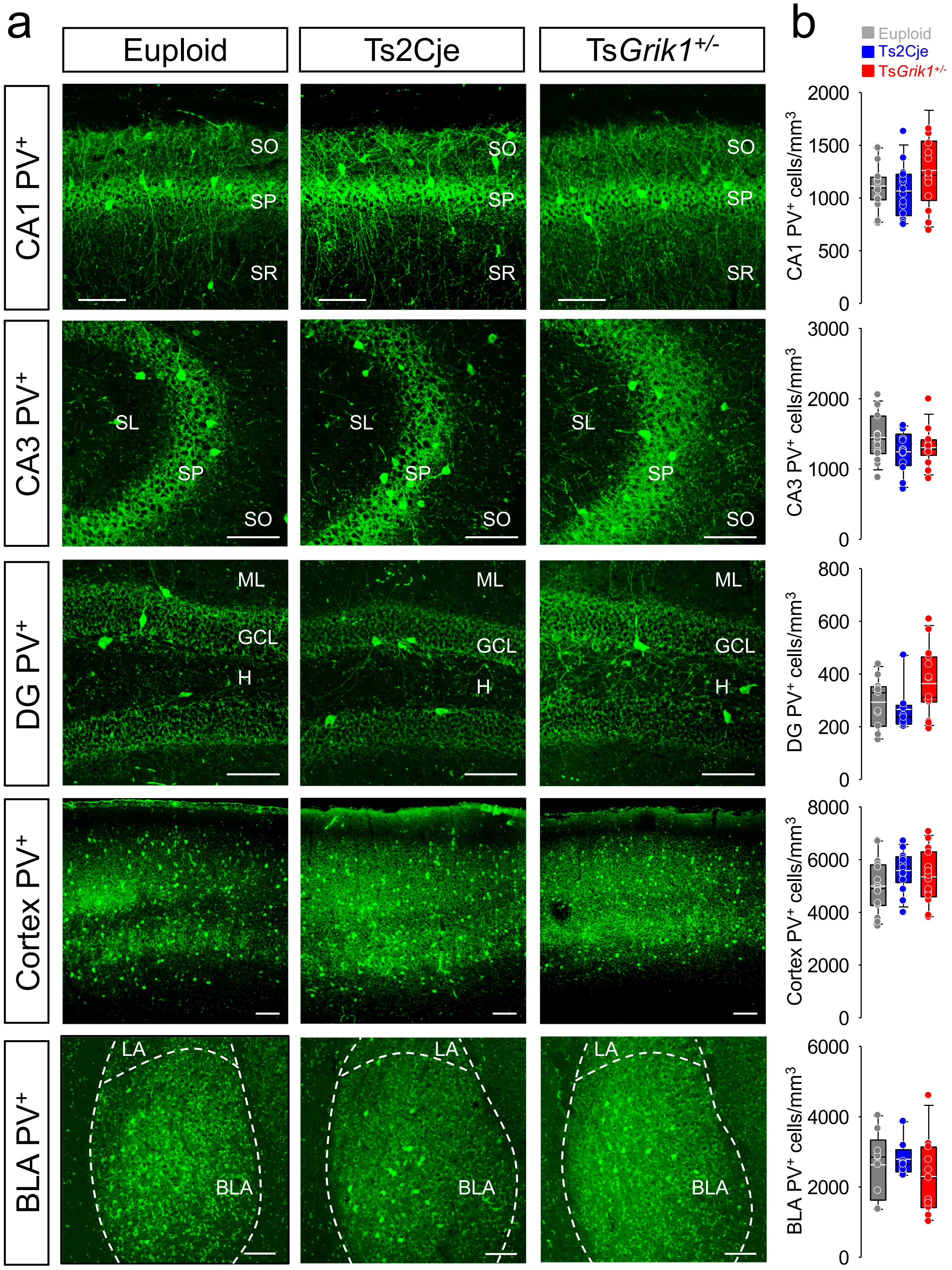

Supplementary Figure 4. Unaltered density of parvalbumin (PV$^+$) interneurons in Ts2Cje and Ts*Grik1*$^{+/-}$ mice. a, Examples of PV$^+$ cells in several brain areas in Ts2Cje and Ts*Grik1*$^{+/-}$ mice. Dashed lines in BLA images label the borders of the region. **b**, Quantifications of the density of PV$^+$ interneurons in hippocampal CA1, CA3, and DG, cortex and BLA of Ts2Cje and Ts*Grik1*$^{+/-}$ mice. SR: stratum radiatum; SP; stratum pyramidale; SO; stratum oriens; ML: molecular layer; GCL: granule cell layer; SL: stratum lucidum; H: hilus; LA: lateral amygdala; BlA: basolateral amygdala. The scale bar corresponds to 100 μm. For detailed data values and statistics see Supplementary Table 1.

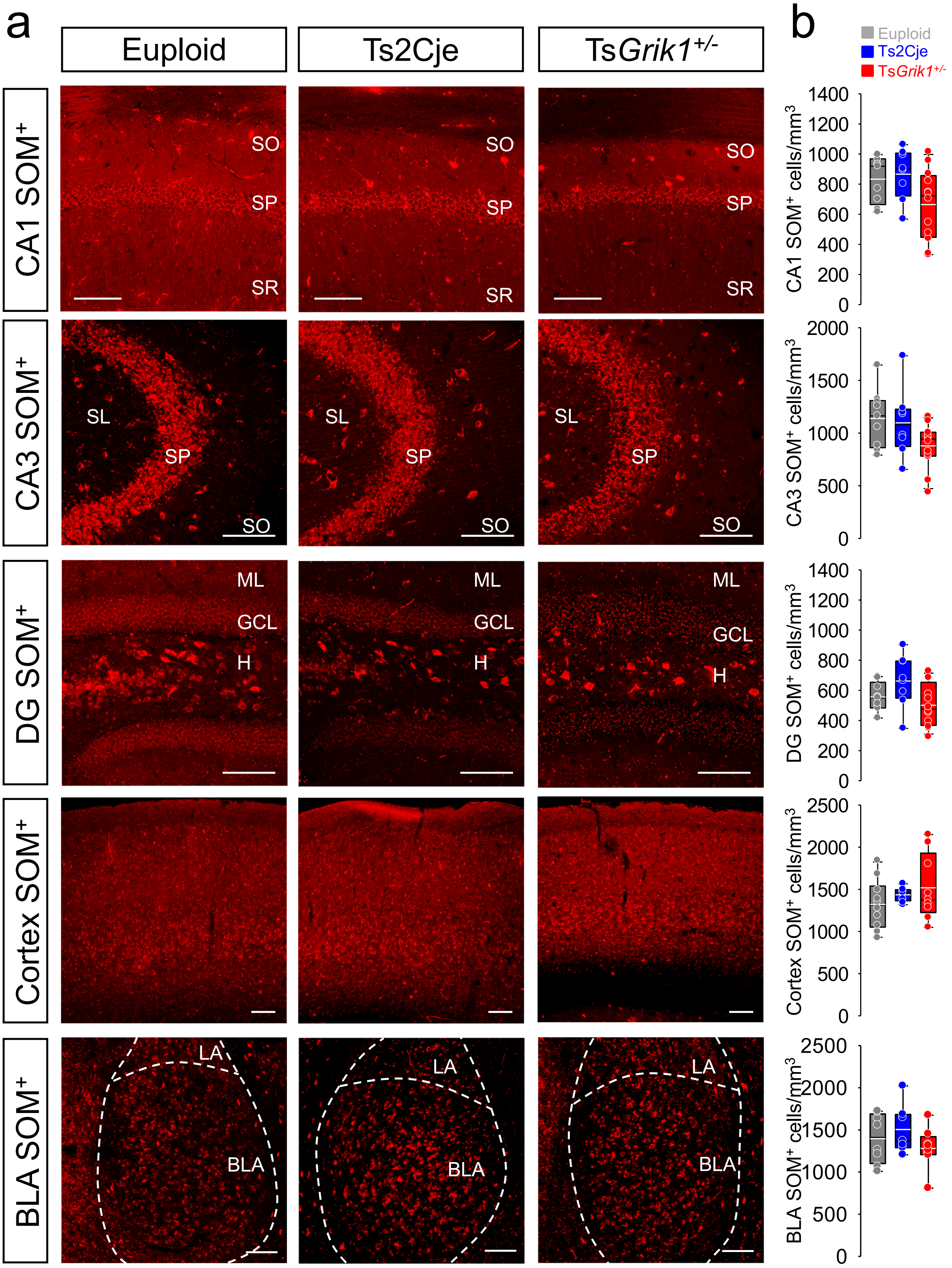

Supplementary Figure 5. Unaltered density of somatostatin (SOM+) interneurons in Ts2Cje and Ts*Grik1*+/- mice. a, Examples of SOM+ cells in several brain areas in Ts2Cje and Ts*Grik1*+/- mice. Dashed lines in BLA images label the borders of the region. **b**, Quantifications of the density of SOM+ interneurons in hippocampal CA1, CA3 and DG, cortex and BLA of Ts2Cje and Ts*Grik1*+/- mice. SR: stratum radiatum; SP; stratum pyramidale; SO; stratum oriens; SL: stratum lucidum; ML: molecular layer; GCL: granule cell layer; H: hilus; LA: lateral amygdala; BLA: basolaterral amygdala. The scale bar corresponds to 100 μm. For detailed data values and statistics see Supplementary Table 1.

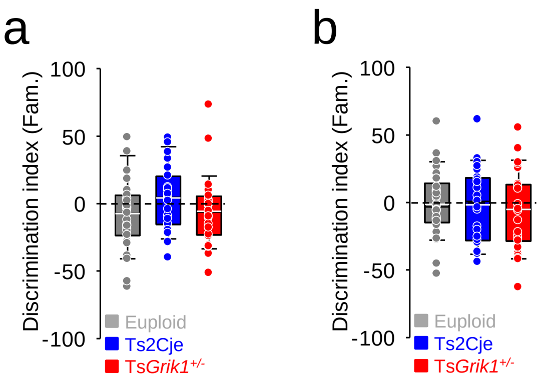

Supplementary Figure 6. Familiarization phase of novel object location and recognition tests. a, Box plot representing the discrimination index at the training phase of the NOL task. Mice from all genotypes explored equally two identical objects placed in training locations in the NOL training phase. **b**, Box plot representing the discrimination index at the training phase of the NOR task. Mice from all genotypes explored equally two identical objects in the NOR training phase. For detailed data values and statistics see Supplementary Table 2.

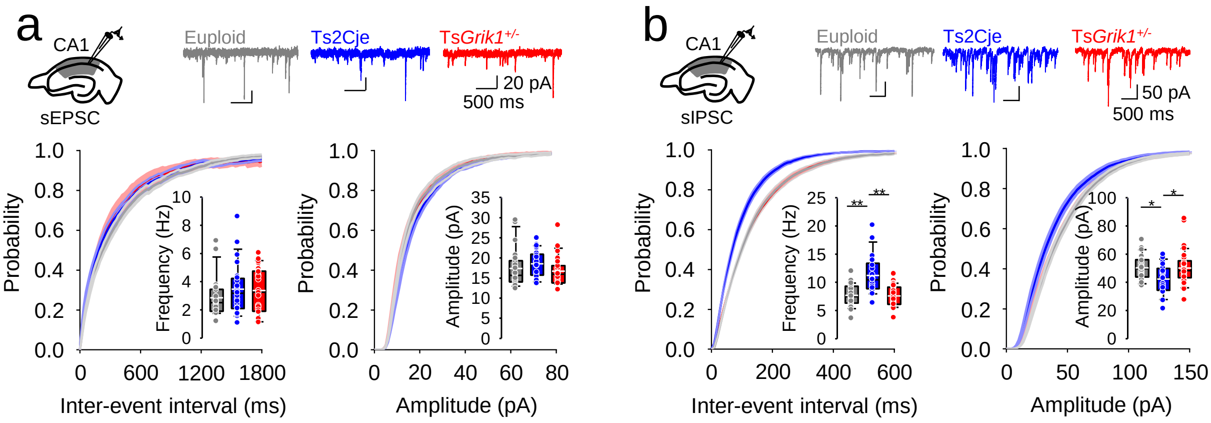

Supplementary Figure 7. *Grik1*-dependent alterations in sIPSCs frequency and amplitude in Ts2Cje mice. a, Basal excitatory synaptic transmission (sEPSCs) in CA1. Top, schematic view of the area recorded and representative traces of sEPSCs in CA1. Bottom, cumulative histograms and box plots showing that frequency and amplitude of sEPSCs in CA1 field was unaltered in Ts2Cje and Ts*Grik1*$^{+/-}$ mice. **b**, Basal inhibitory synaptic transmission (sIPSCs) in CA1. Top, schematic view of the area recorded and representative traces of sIPSCs in CA1. Bottom, plots like in **a** showing that sIPSCs have increased frequency and reduced amplitude in CA1 of Ts2Cje mice, which were restored to euploid levels in Ts*Grik1*$^{+/-}$ littermates. For detailed data values and statistics see Supplementary Table 2. $*p < 0.05$, $***p < 0.005$.

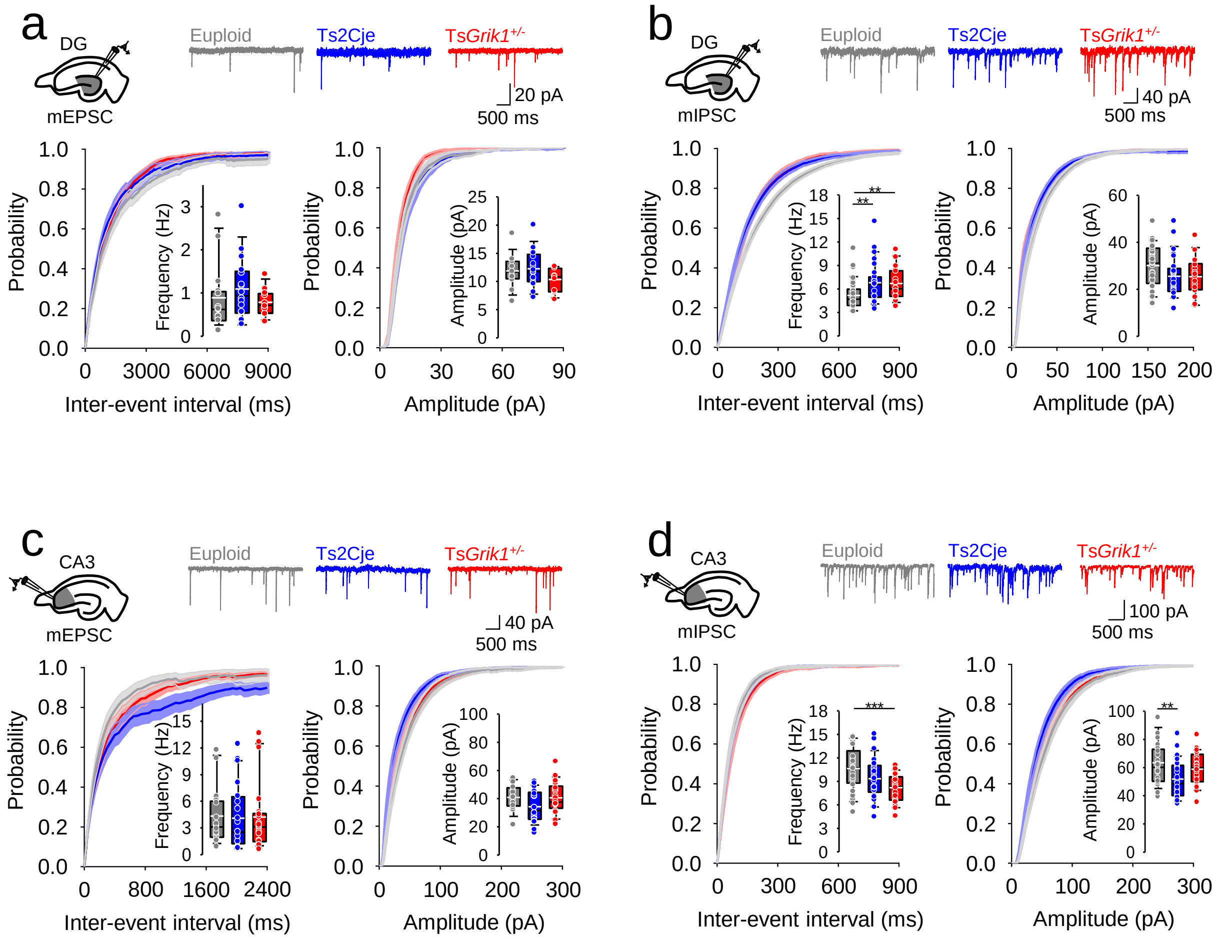

Supplementary Figure 8. *Grik1*-independent basal synaptic transmission phenotypes in DG and CA3 of Ts2Cje mice. a, Basal excitatory and inhibitory synaptic transmission (mEPSCs, mIPSCs) were recorded in DG granule cells (**a, b**) and CA3 pyramidal neurons (**c,d**)**.** At the top of each panel a schematic view of the area recorded and representative traces of mEPSCs are show. At the bottom cumulative histograms and box plots showing that the frequency and amplitude of mEPSCs were unaltered in DG and CA3 of Ts2Cje and Ts*Grik1*$^{+/-}$ mice (**a, c**) whilst the basal inhibitory synaptic transmission (mIPSCs) was altered in both DG and CA3 of Ts2Cje but not recovered in Ts*Grik1*$^{+/-}$. For detailed data values and statistics see Supplementary Table 2. *$p < 0.05$, ***$p < 0.005$.

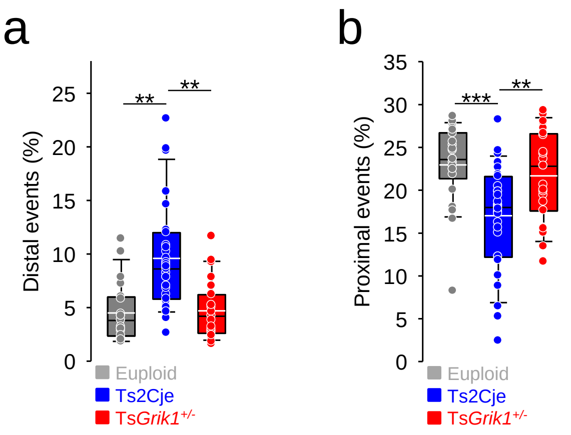

Supplementary Figure 9. *Grik1*-dependent bidirectional alteration in proportion of distal and proximal spontaneous IPSC (mIPSC) in CA1 of Ts2Cje mice. a, Box plot representing increased proportion of slow and small (i.e. distal) mIPSCs in Ts2Cje mice. **b**, Box plot showing reduced frequency of fast and large (i.e. proximal) events in Ts2Cje mice. For detailed data values and statistics see Supplementary Table 3. *$p < 0.05$, ***$p < 0.005$.

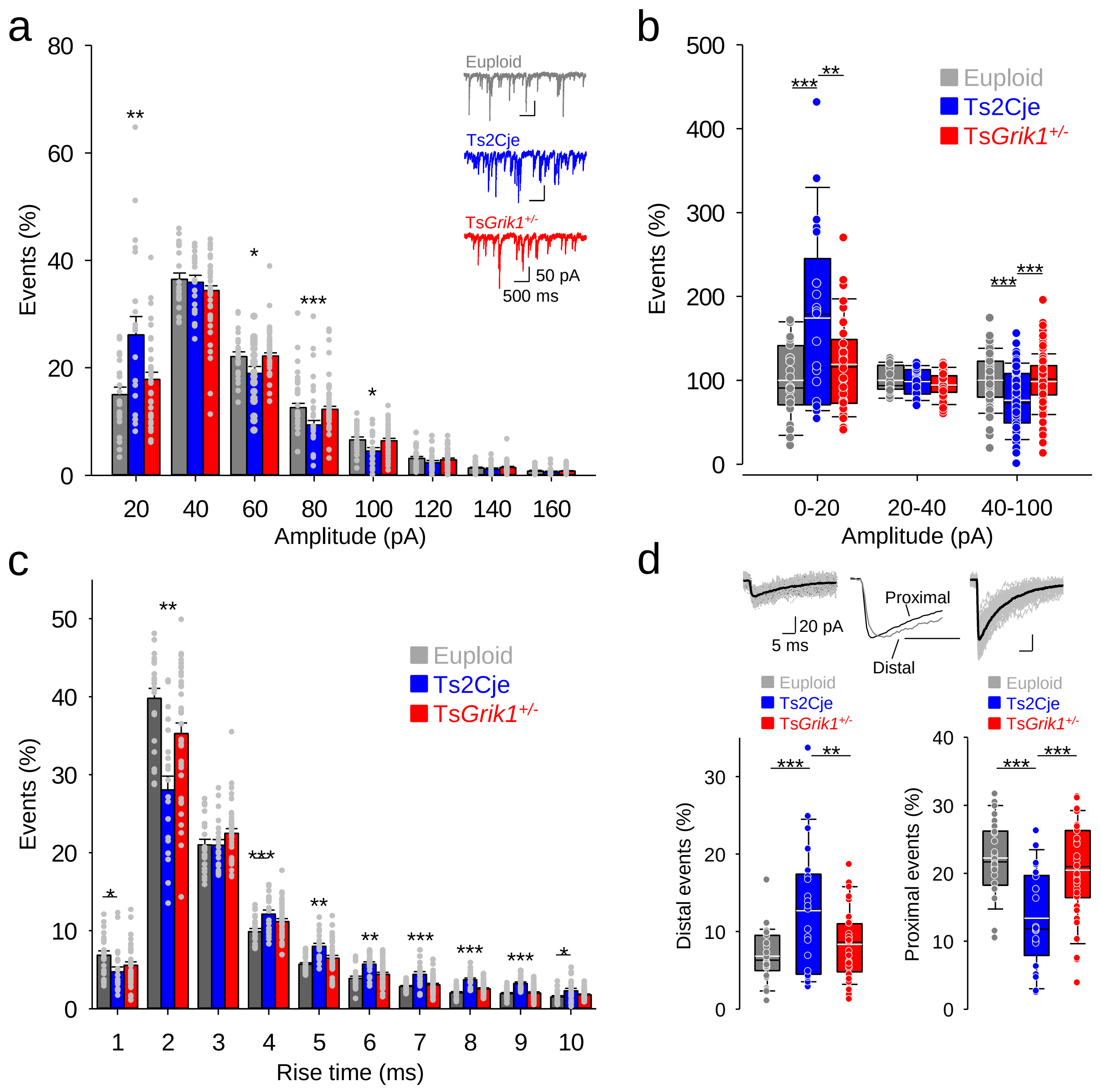

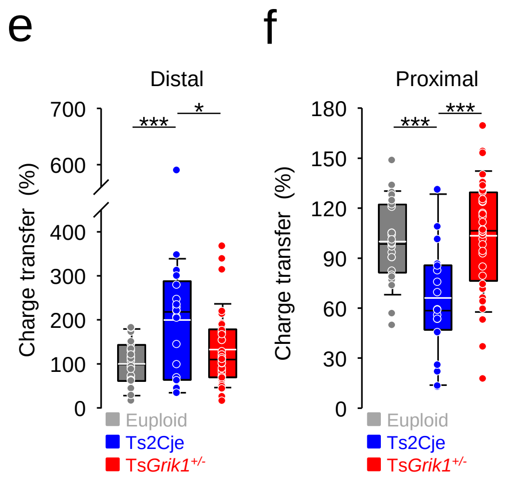

Supplementary Figure 10. *Grik1* triplication causes bidirectional alterations in hippocampal GABAergic transmission (sIPSCs) in Ts2Cje mice. a, Frequency histogram of CA1 pyramidal neurons spontaneurs inhibitory postsynaptic currents (sIPSCs) sorted in 20 pA bins to assess the relative proportion of small and large events across genotypes. Increased proportion of small sIPSCs (<20 pA) and decreased proportion of large events (40-100 pA) in Ts2Cje mice are restored upon normalization of *Grik1* dosage. **b,** Sorting of sIPSCs in small, medium-sized and large categories. Box plot illustrates increased and decreased proportion of small and large events, respectively, in Ts2Cje mice compared to euploid and Ts*Grik1*$^{+/-}$ littermates **c**, Frequency histogram of sIPSCs sorted according to their rise time (1 ms bins). *Grik1*-dependent decrease and increase of fast and slow sIPSCs, respectively, in CA1 region of Ts2Cje mice. **d**, Top, insets showing example traces of distal (left), proximal (right) and superimposed normalized distal and proximal events (center). Bottom, left panel, box plot showing increased proportion of distal (small and slow) sIPSCs in Ts2Cje mice was restored in Ts*Grik1*$^{+/-}$ mice. Right panel, box plot representing *Grik1*-dependent reduction in proportion of proximal (large and fast) sIPSCs in Ts2Cje mice. **e**, Box plots showing quantification of charge transfer at distal regions of the somatodendritic axis in CA1 pyramidal neurons. Left, increased charge carried by distal sIPSCs in Ts2Cje mice was recovered to euploid levels upon normalization of *Grik1* dosage. **f**, Box plot showing decreased charge transfer carried by proximal sIPSCs in Ts2Cje mice in comparison to euploid and Ts*Grik1*$^{+/-}$ littermates. In bar plots, error bars represent the s.e.m. For detailed data values and statistics see Supplementary Table 3. *$p < 0.05$, ***$p < 0.005$.

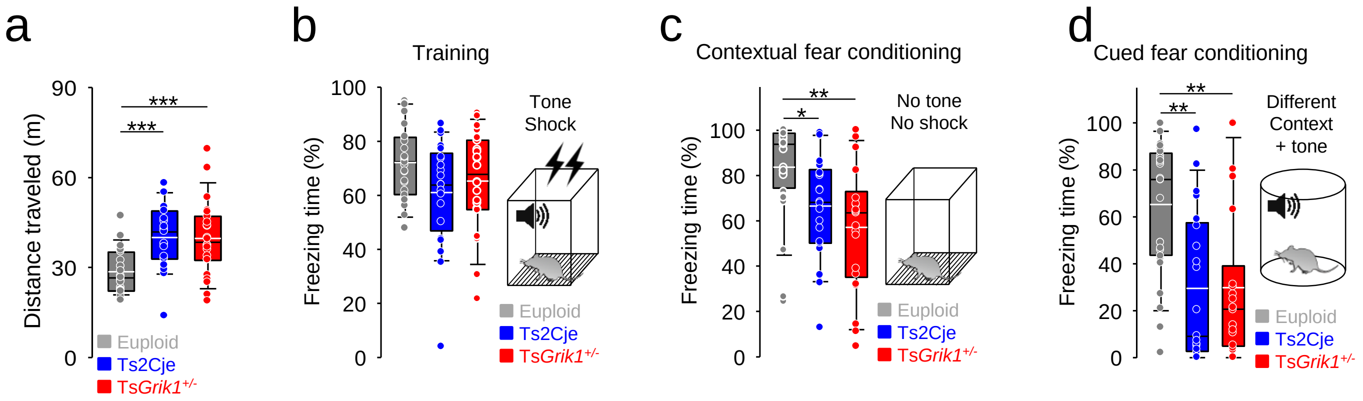

Supplementary Figure 11. Increased locomotion and decreased fear conditioning in Ts2Cje and Ts*Grik1*$^{+/-}$ mice. a, Box plot representing the distance traveled in the elevated plus maze. As in Fig. 5a, Ts2Cje and Ts*Grik1*$^{+/-}$ mice showed increased distance traveled in the elevated plus maze. **b**, Training session of the fear conditioning paradigm. Left, box plot illustrating freezing after application of an electric shock. Right, schematic view of the training paradigm. **c**, Left, Box plot showing reduced freezing in Ts2Cje and Ts*Grik1*$^{+/-}$ mice at the contextual phase of fear conditioning test. Right, schematic view of the contextual fear conditioning paradigm. **d**, Left, Box plot illustrating reduced freezing upon exposure to the CS in Ts2Cje and Ts*Grik1*$^{+/-}$ mice. Right, experimental paradigm for the cued fear conditioning test. For detailed data values and statistics see Supplementary Table 5.

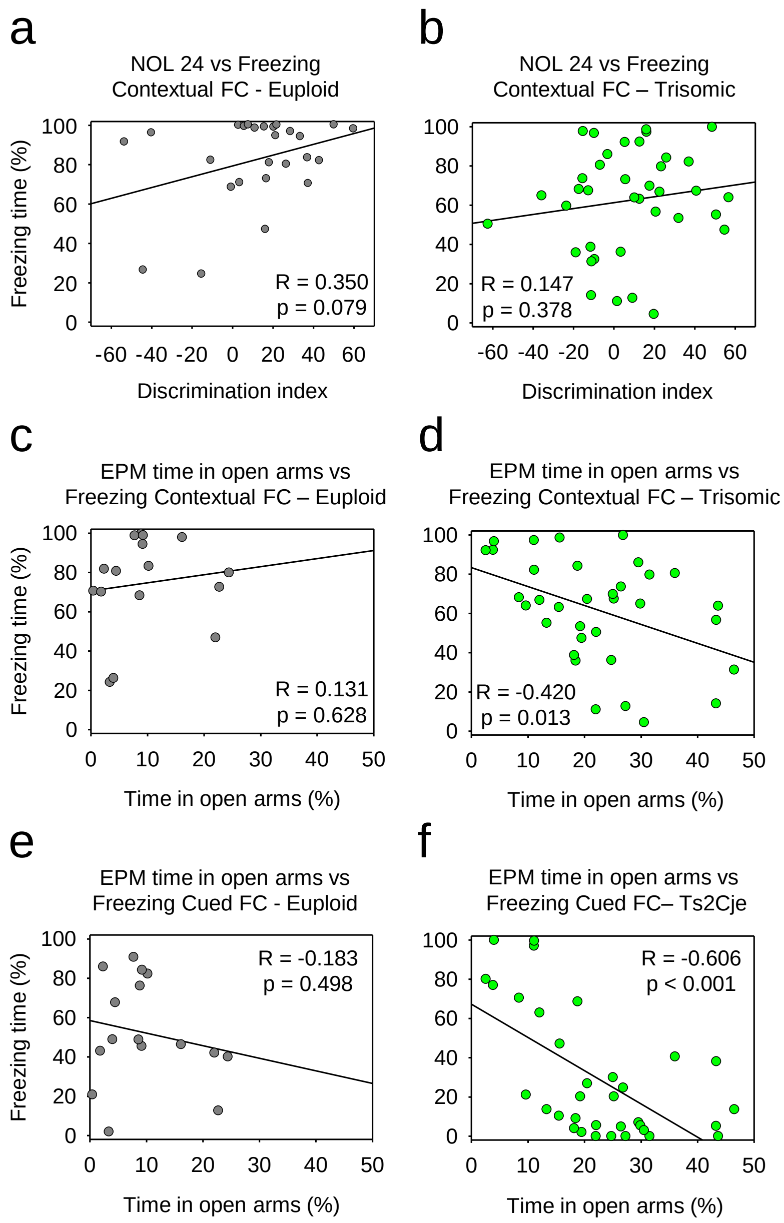

Supplementary Figure 12. Fear conditioning performance is influenced by anxiety levels in trisomic mice. Discrimination indexes in novel object location (NOL) tests were plotted vs. freezing times observed for each animal in the contextual phase of fear conditioning (FC) tests (**a**). Scatter plot show partial correlation between performance in NOL test at 24h and freezing time (%) in euploid mice. **b**, the same plot evidences absence of correlation between these two parameters in trisomic (Ts2Cje + Ts*Grik1*$^{+/-}$) mice. **c, d,** The time spent in the open arms of elevated plus maze (EPM) were plotted vs the freezing times (%) in contextual FC in euploid (**c**) and trisomic (**d**) mice. **e**, **f**, the time in open arms were plotted vs the freezing time (%) in cued FC in euploid (**e**) trisomic mice (**f**). Note how the performance in FC tests became dependent of levels anxiety (time in open arms) in trisomic mice. For detailed data values and statistics see Supplementary Table 5.
