# Supplementary material for: Unbalanced dendritic inhibition of CA1 neurons drives spatial-memory deficits in the Ts2Cje Down syndrome model
Source: Nat Commun. 2019 Nov 1;10:4991. doi: 10.1038/s41467-019-13004-9 (PMC6825203; doi:10.1038/s41467-019-13004-9)
Supplement: Supplementary file 3 — Description of Additional Supplementary Files [file 41467_2019_13004_MOESM3_ESM.docx]

Description of Supplementary Data 1

**Supplementary Table 1.** Detailed values and statistical analyses of Grik1 mRNA levels and GluK1 currents in Ts2Cje mice (Suppl. Fig. 1), mice weights and CA1 pyramidal cell membrane parameters (Suppl. Fig. 2), hot plate and rotarod tests (Suppl. Fig. 3) and PV+ and SOM+ interneurons density (Suppl Figs. 4 and 5).

**Supplementary Table 2.** Detailed values and statistical analyses of Grik1 mRNA levels normalization in hippocampus (Fig. 1), NOL and NOR tests (Fig. 1 and Suppl. Fig. 6) and basal excitatory and inhibitory synaptic transmission in CA1, CA3 and DG (Fig. 1 and Suppl. Figs. 7 and 8).

**Supplementary Table 3.** Detailed values and statistical analyses of m/sIPSCs bidirectional alterations (Fig.3 and Suppl. Fig. 9 and 10) and PPR of eIPSCs (Fig. 4).

**Supplementary Table 4.** Detailed values and statistical analyses of synaptic plasticity phenotypes (Fig. 2).

**Supplementary Table 5.** Detailed values and statistical analyses of open field, elevated plus maze and fear conditioning (Fig. 5 and Suppl. Fig. 11 and 12) and basal synaptic transmission in BLA (Fig. 5).
